# Crystal structure of bis­(μ_2_-tetra­bromo­phthalato-κ^2^ *O* ^1^:*O* ^2^)bis[aqua(*N*,*N*,*N*′,*N*′-tetra­methyl­ethane-1,2-di­amine-κ^2^ *N*,*N*′)copper(II)]

**DOI:** 10.1107/S2056989015015194

**Published:** 2015-08-22

**Authors:** Luis Manuel Tobón-Trujillo, Luis Felipe Villanueva-Sánchez, Diego Martínez-Otero, Alejandro Dorazco-González

**Affiliations:** a Centro Conjunto de Investigación en Química Sustentable UAEM-UNAM, Instituto de Química, Universidad Nacional Autónoma de México, Carretera Toluca-Atlacomulco, Km 14.5 CP 50200 Toluca, Estado de México, México

**Keywords:** crystal structure, copper(II) complex, tetra­methyl­ethane-1,2-di­amine, tetra­bromo­phthalate anion, hydrogen bonding

## Abstract

In the title complex, [Cu_2_(C_8_Br_4_O_4_)_2_(C_6_H_16_N_2_)_2_(H_2_O)_2_], the Cu^II^ cation is chelated by a tetra­methyl­ethane-1,2-di­amine ligand and coordinated by a water mol­ecule as well as bridged by two tetra­bromo­phthalate anions in a distorted O_3_N_2_ trigonal–bipyramidal geometry. The two symmetry-related tetra­bromo­phthalate anions bridge the two Cu^II^ cations, forming a centrosymmetric dinuclear complex in which the Cu⋯Cu separation is 5.054 (2) Å. Intra­molecular classic O—H⋯O hydrogen bonds and weak C—H⋯O hydrogen bonds occur in the dinuclear mol­ecule. In the crystal, the mol­ecules are linked by weak C—H⋯Br and C—H⋯O inter­actions into supra­molecular chains propagating along the *b*-axis direction.

## Related literature   

For the crystal structures of related copper(II) complexes with tetramethylethylen-1,2-diamine and carboxyl­ate ligands; see: Ene *et al.* (2009 ▸); Dorazco-González *et al.* (2013 ▸); Liang *et al.* (2004 ▸). For the synthesis of coordination compounds with one-dimensional polymeric structures, see: Hong & You (2004 ▸); Colacio *et al.* (2009 ▸); Rodpun *et al.* (2015 ▸); Yang *et al.* (2002 ▸). For their magnetic properties, see: Ene *et al.* (2009 ▸); Kozlevčar *et al.* (2004 ▸). For supra­molecular polymorphism, see: Dorazco-González *et al.* (2013 ▸); Stibrany *et al.* (2009 ▸); Aakeröy *et al.* (2003 ▸); Valdés-Martínez *et al.* (1993 ▸); Julve *et al.* (1984 ▸). For mol­ecular recognition and sensing; see: Dorazco-González & Yatsimirsky (2010 ▸); Mendy *et al.* (2010 ▸).
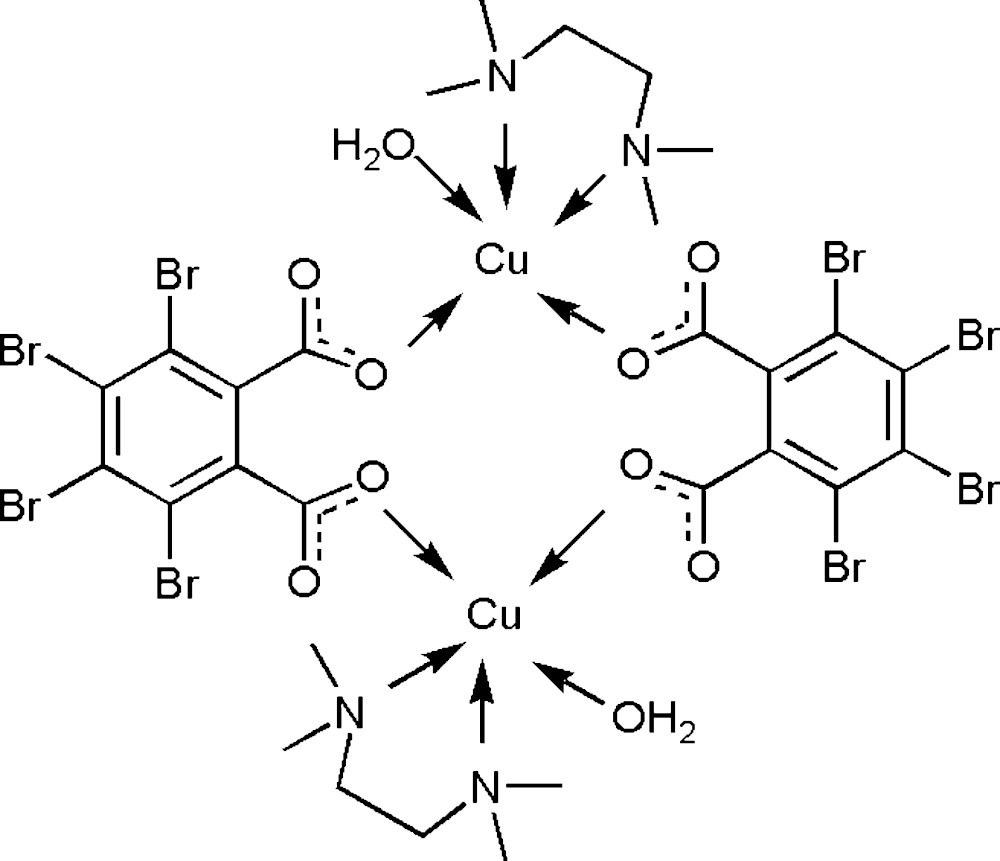


## Experimental   

### Crystal data  

[Cu_2_(C_8_Br_4_O_4_)_2_(C_6_H_16_N_2_)_2_(H_2_O)_2_]
*M* *_r_* = 1354.97
Monoclinic, 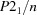

*a* = 9.0961 (2) Å
*b* = 9.2281 (2) Å
*c* = 24.4026 (7) Å
β = 95.4910 (6)°
*V* = 2038.95 (9) Å^3^
*Z* = 2
Mo *K*α radiation
μ = 8.94 mm^−1^
*T* = 100 K
0.25 × 0.15 × 0.08 mm

### Data collection  

Bruker APEXII CCD diffractometer
Absorption correction: multi-scan (*SADABS*; Bruker, 2012 ▸) *T*_min_ = 0.442, *T*_max_ = 0.745
17394 measured reflections
3739 independent reflections
3547 reflections with *I* > 2σ(*I*)
*R*_int_ = 0.019

### Refinement  

*R*[*F* ^2^ > 2σ(*F* ^2^)] = 0.016
*wR*(*F* ^2^) = 0.039
*S* = 1.07
3739 reflections
245 parameters
2 restraints
H atoms treated by a mixture of independent and constrained refinement
Δρ_max_ = 0.80 e Å^−3^
Δρ_min_ = −0.41 e Å^−3^

### 

Data collection: *APEX2* (Bruker, 2012 ▸); cell refinement: *SAINT* (Bruker, 2012 ▸); data reduction: *SAINT*; program(s) used to solve structure: *SHELXS97* (Sheldrick 2008 ▸); program(s) used to refine structure: *SHELXL2013* (Sheldrick, 2008 ▸); molecular graphics: *SHELXTL* (Sheldrick, 2008 ▸); software used to prepare material for publication: *SHELXTL*.

## Supplementary Material

Crystal structure: contains datablock(s) I, New_Global_Publ_Block. DOI: 10.1107/S2056989015015194/xu5866sup1.cif

Structure factors: contains datablock(s) I. DOI: 10.1107/S2056989015015194/xu5866Isup2.hkl

**Fig. 1.**
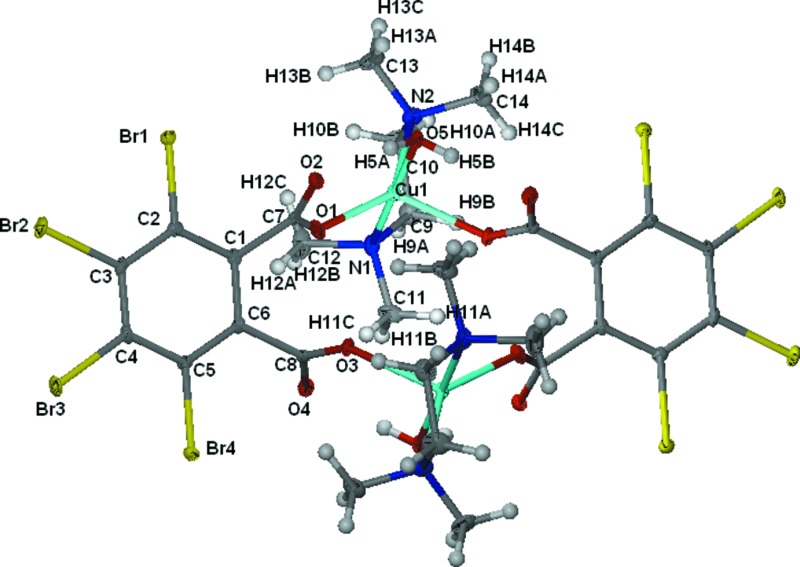
The structure with displacement ellipsoids drawn at the 30% probability level and H atoms shown as small spheres of arbitrary radii.

. DOI: 10.1107/S2056989015015194/xu5866fig1.tif
The structure with displacement ellipsoids drawn at the 30% probability level and H atoms shown as small spheres of arbitrary radii.

**Fig. 2.**
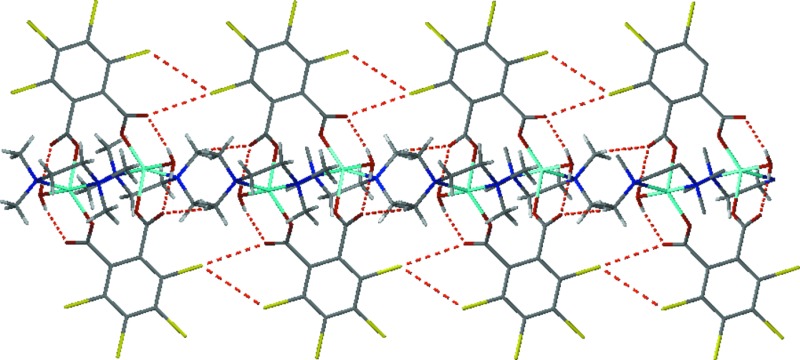
View of interactions in the crystal.

. DOI: 10.1107/S2056989015015194/xu5866fig2.tif
View of inter­actions in the crystal.

CCDC reference: 1418832

Additional supporting information:  crystallographic information; 3D view; checkCIF report

## Figures and Tables

**Table 1:** Hydrogen-bond geometry (, )

*D*H*A*	*D*H	H*A*	*D* *A*	*D*H*A*
O5H5*A*O2	0.81(2)	1.87(2)	2.649(2)	161(2)
O5H5*B*O4^i^	0.82(2)	1.83(2)	2.630(2)	167(3)
C10H10*A*Br4^ii^	0.99	2.84	3.729(3)	150
C11H11*C*O4	0.98	2.40	3.376(3)	179
C13H13*A*O4^iii^	0.98	2.58	3.506(3)	158

Symmetry codes: (i) 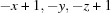; (ii) 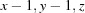; (iii) 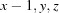.

## supplementary crystallographic information

### S1. Introduction

The self-assembly of metal complexes with benzene-multi­carboxyl­ate ligands remains an active area in coordination chemistry especially with copper due to the very wide structural diversity and inter­esting properties in magnetism, host-guest systems, porous material (Ene *et al.*, 2009; Dorazco-González *et al.*, 2013; Liang *et al.*, 2004). Dinuclear copper(II) compounds have been used in magnetism, as biomimetic active sites in bioinorganic chemistry and in the design and synthesis of metallic networks (Lu *et al.*, 2004). Herein we present a dinuclear copper complex synthesised by self-assembly between copper perchlorate, a aliphatic di­amine (tmen) and a bulky benzendi­carboxyl­ate (tetra­bromo­phthalate). The tittle compound represents the first example of copper complex with tetra­bromo­phthalate.

### S2. Experimental

Compound. Cu(ClO_4_)_2_.6H_2_O (0.1 mmol, 0.037 g) was added directly to a solution of tmen (0.1 mmol) in metanol-water solution 1:1 (8 mL). Then the dianion of tetra­bromo­phthalic acid (0.1 mmol, 0.055 g) in methanol-water solution 1:1 again (12 mL) was slowly added with stirring at room temperature, and a slight warming at 50 °C for 3 minutes.

### S2.1. Synthesis and crystallization

Blue deep suitable crystals for diffraction X-ray were grown directly from solution by slow evaporation during 5 days. IR(KBr pellet), 3012 (w), 2971 (w), 2947 (w), 1741 (s), 1606 (m), 1396 (m), 1504 (w), 1459 (w), 1371 (s), 1315 (m), 1213 (m), 1096 (w), 1018 (w), 951 (w), 867 (w), 805 (w), 765 (w), 733 (w), 558 (w), 525 (w).

### S2.2. Refinement

Water H atoms were placed in a difference Fourier map and positional parameters were refined, U_iso_(H) = 1.2U_eq_(O). Other H atoms were placed in calculated positions with C—H = 0.98–0.99 Å and refined in riding mode, U_iso_(H) = 1.2U_eq_(C) for methyl­ene H atoms and 1.5U_eq_(C) for methyl H atoms.

### S3. Results and discussion

The reaction between the aqua-complex [Cu(tmen)(H_2_O)_x_](ClO_4_)_2_ and potassium salt of tetra­bromo­phthalate in mixture ethanol gives the title compound in good yield (>94 %) as blue suitable crystals for X ray diffraction. The single-crystal X-ray analysis reveals that the compound is a neutral dinuclear centro-symmetric copper (II) complex which crystallizes in monoclinic crystal system, space group P21/n (Figure 1). The asymmetric unit contains a five-coordinate copper atom [Cu(N_2_O_3_)] with two sites occupied by di­amine and three sites by oxygen atoms from two carboxyl­ate groups and one molecule of water. The Addison tau-parameter has been used to describe the distortion around coordination geometry, τ = (difference between two largest angles/60 for five-coordinated metal centers allows the distinction between trigonal-bipyramidal (ideally τ = 1) and square-pyramidal (ideally τ = 0). In this context, the coordination geometry of complex is distorted trigonal-bypiramide, τ = 0.68. The distance Cu ··· Cu is 5.054 (2) Å and a macrocycle is formed by 14 atoms containing two TBr-phthalate-bridge ligands. The combination of copper(II) with multi-carb­oxy­lic acids has formed one of the largest subgroups in metal-organic compounds and despite this there are few examples of coordination complex with 1,2,3,4,-tetra­halogenated benzenes among these only with tetra­chloro­phthalate have been reported (Hong & You, 2004; Yang *et al.*, 2002). The present compound represents the first example with tetra­bromo­phthalate.

### Crystal data

**Table:**

[Cu_2_(C_8_Br_4_O_4_)_2_(C_6_H_16_N_2_)_2_(H_2_O)_2_]	*F*(000) = 1300
*M**_r_* = 1354.97	*D*_x_ = 2.207 Mg m^−^^3^
Monoclinic, *P*2_1_/*n*	Mo *K*α radiation, λ = 0.71073 Å
*a* = 9.0961 (2) Å	Cell parameters from 9880 reflections
*b* = 9.2281 (2) Å	θ = 2.4–25.7°
*c* = 24.4026 (7) Å	µ = 8.94 mm^−^^1^
β = 95.4910 (6)°	*T* = 100 K
*V* = 2038.95 (9) Å^3^	Prism, blue
*Z* = 2	0.25 × 0.15 × 0.08 mm

### Data collection

**Table:**

Bruker APEXII CCD diffractometer	3739 independent reflections
Radiation source: Incoatec ImuS	3547 reflections with *I* > 2σ(*I*)
Mirrors monochromator	*R*_int_ = 0.019
ω scans	θ_max_ = 25.4°, θ_min_ = 2.3°
Absorption correction: multi-scan (*SADABS*; Bruker, 2012)	*h* = −10→10
*T*_min_ = 0.442, *T*_max_ = 0.745	*k* = −11→11
17394 measured reflections	*l* = −29→29

### Refinement

**Table:**

Refinement on *F*^2^	2 restraints
Least-squares matrix: full	Hydrogen site location: inferred from neighbouring sites
*R*[*F*^2^ > 2σ(*F*^2^)] = 0.016	H atoms treated by a mixture of independent and constrained refinement
*wR*(*F*^2^) = 0.039	*w* = 1/[σ^2^(*F*_o_^2^) + (0.0162*P*)^2^ + 2.4857*P*] where *P* = (*F*_o_^2^ + 2*F*_c_^2^)/3
*S* = 1.07	(Δ/σ)_max_ = 0.002
3739 reflections	Δρ_max_ = 0.80 e Å^−^^3^
245 parameters	Δρ_min_ = −0.41 e Å^−^^3^

### Special details

**Table:**

Geometry. All e.s.d.'s (except the e.s.d. in the dihedral angle between two l.s. planes) are estimated using the full covariance matrix. The cell e.s.d.'s are taken into account individually in the estimation of e.s.d.'s in distances, angles and torsion angles; correlations between e.s.d.'s in cell parameters are only used when they are defined by crystal symmetry. An approximate (isotropic) treatment of cell e.s.d.'s is used for estimating e.s.d.'s involving l.s. planes.

### Fractional atomic coordinates and isotropic or equivalent isotropic displacement parameters (Å^2^)

**Table:**

	*x*	*y*	*z*	*U*_iso_*/*U*_eq_	
Br1	0.34950 (2)	0.30336 (3)	0.71377 (2)	0.01607 (6)	
Br2	0.62992 (3)	0.47283 (3)	0.78249 (2)	0.02118 (6)	
Br3	0.95418 (3)	0.47738 (3)	0.73268 (2)	0.01934 (6)	
Br4	1.00627 (2)	0.27574 (3)	0.62483 (2)	0.01671 (6)	
Cu1	0.32727 (3)	−0.12890 (3)	0.55897 (2)	0.01122 (6)	
O1	0.45257 (16)	0.02014 (16)	0.59851 (6)	0.0143 (3)	
O2	0.32220 (16)	0.22748 (16)	0.58559 (6)	0.0135 (3)	
O3	0.65378 (16)	0.19336 (16)	0.52428 (6)	0.0132 (3)	
O4	0.79787 (17)	0.02815 (16)	0.57131 (6)	0.0144 (3)	
O5	0.17319 (17)	0.01324 (17)	0.53376 (6)	0.0133 (3)	
H5A	0.205 (3)	0.091 (2)	0.5454 (10)	0.016*	
H5B	0.179 (3)	0.014 (3)	0.5006 (7)	0.016*	
N1	0.4780 (2)	−0.2831 (2)	0.58485 (7)	0.0153 (4)	
N2	0.1667 (2)	−0.2787 (2)	0.57861 (8)	0.0180 (4)	
C1	0.5567 (2)	0.2309 (2)	0.63834 (8)	0.0102 (4)	
C2	0.5382 (2)	0.3046 (2)	0.68693 (8)	0.0108 (4)	
C3	0.6563 (2)	0.3755 (2)	0.71631 (8)	0.0119 (4)	
C4	0.7951 (2)	0.3723 (2)	0.69622 (8)	0.0123 (4)	
C5	0.8155 (2)	0.2926 (2)	0.64905 (8)	0.0114 (4)	
C6	0.6979 (2)	0.2221 (2)	0.62005 (8)	0.0101 (4)	
C7	0.4303 (2)	0.1547 (2)	0.60456 (8)	0.0104 (4)	
C8	0.7190 (2)	0.1388 (2)	0.56745 (8)	0.0112 (4)	
C9	0.3941 (3)	−0.4200 (2)	0.57551 (10)	0.0206 (5)	
H9A	0.4525	−0.5017	0.5925	0.025*	
H9B	0.3757	−0.4388	0.5355	0.025*	
C10	0.2483 (3)	−0.4091 (3)	0.60056 (11)	0.0267 (6)	
H10A	0.1885	−0.4970	0.5915	0.032*	
H10B	0.2665	−0.4022	0.6411	0.032*	
C11	0.6075 (3)	−0.2849 (3)	0.55284 (11)	0.0221 (5)	
H11A	0.5747	−0.2979	0.5137	0.033*	
H11B	0.6728	−0.3651	0.5655	0.033*	
H11C	0.6611	−0.1931	0.5581	0.033*	
C12	0.5316 (3)	−0.2652 (3)	0.64386 (10)	0.0295 (6)	
H12A	0.5925	−0.1776	0.6485	0.044*	
H12B	0.5908	−0.3497	0.6564	0.044*	
H12C	0.4470	−0.2564	0.6657	0.044*	
C13	0.0750 (3)	−0.2171 (3)	0.61946 (10)	0.0270 (6)	
H13A	0.0211	−0.1327	0.6036	0.040*	
H13B	0.1384	−0.1874	0.6523	0.040*	
H13C	0.0045	−0.2903	0.6297	0.040*	
C14	0.0693 (3)	−0.3163 (3)	0.52836 (10)	0.0244 (5)	
H14A	0.0251	−0.2277	0.5119	0.037*	
H14B	−0.0091	−0.3815	0.5381	0.037*	
H14C	0.1275	−0.3644	0.5019	0.037*	

### Atomic displacement parameters (Å^2^)

**Table:**

	*U*^11^	*U*^22^	*U*^33^	*U*^12^	*U*^13^	*U*^23^
Br1	0.01284 (11)	0.02352 (12)	0.01199 (11)	0.00231 (9)	0.00185 (8)	−0.00263 (9)
Br2	0.02459 (13)	0.02531 (13)	0.01399 (11)	−0.00390 (10)	0.00359 (9)	−0.01160 (9)
Br3	0.02013 (12)	0.02323 (13)	0.01411 (11)	−0.01075 (9)	−0.00115 (8)	−0.00597 (9)
Br4	0.01220 (11)	0.02355 (13)	0.01461 (11)	−0.00571 (9)	0.00253 (8)	−0.00292 (9)
Cu1	0.01308 (13)	0.00842 (13)	0.01167 (13)	−0.00056 (10)	−0.00133 (10)	−0.00121 (10)
O1	0.0144 (8)	0.0097 (8)	0.0178 (8)	−0.0008 (6)	−0.0039 (6)	−0.0018 (6)
O2	0.0123 (8)	0.0125 (8)	0.0151 (8)	0.0011 (6)	−0.0019 (6)	−0.0023 (6)
O3	0.0172 (8)	0.0142 (8)	0.0080 (7)	0.0012 (6)	0.0002 (6)	−0.0017 (6)
O4	0.0168 (8)	0.0129 (8)	0.0130 (7)	0.0032 (6)	−0.0005 (6)	−0.0012 (6)
O5	0.0160 (8)	0.0127 (8)	0.0109 (7)	−0.0013 (6)	−0.0006 (6)	−0.0043 (6)
N1	0.0210 (10)	0.0108 (9)	0.0136 (9)	−0.0004 (8)	−0.0011 (7)	0.0003 (7)
N2	0.0196 (10)	0.0167 (10)	0.0185 (10)	−0.0029 (8)	0.0052 (8)	0.0036 (8)
C1	0.0138 (10)	0.0060 (10)	0.0103 (10)	0.0006 (8)	−0.0015 (8)	0.0030 (8)
C2	0.0125 (10)	0.0084 (10)	0.0116 (10)	0.0017 (8)	0.0023 (8)	0.0020 (8)
C3	0.0190 (11)	0.0095 (10)	0.0070 (10)	0.0005 (9)	0.0003 (8)	−0.0012 (8)
C4	0.0160 (11)	0.0100 (10)	0.0100 (10)	−0.0037 (9)	−0.0040 (8)	0.0007 (8)
C5	0.0131 (10)	0.0115 (10)	0.0100 (10)	−0.0017 (8)	0.0032 (8)	0.0027 (8)
C6	0.0148 (11)	0.0084 (10)	0.0069 (10)	0.0001 (8)	−0.0003 (8)	0.0031 (8)
C7	0.0122 (10)	0.0117 (11)	0.0074 (9)	−0.0017 (8)	0.0022 (8)	−0.0004 (8)
C8	0.0104 (10)	0.0117 (11)	0.0116 (10)	−0.0047 (9)	0.0025 (8)	−0.0009 (8)
C9	0.0255 (13)	0.0097 (11)	0.0260 (13)	−0.0008 (10)	0.0003 (10)	0.0021 (9)
C10	0.0288 (14)	0.0165 (12)	0.0357 (15)	−0.0019 (11)	0.0077 (11)	0.0117 (11)
C11	0.0169 (12)	0.0180 (12)	0.0311 (14)	0.0039 (10)	0.0009 (10)	0.0069 (10)
C12	0.0426 (16)	0.0238 (14)	0.0194 (13)	0.0077 (12)	−0.0113 (11)	0.0013 (10)
C13	0.0276 (14)	0.0328 (15)	0.0228 (13)	0.0022 (12)	0.0140 (11)	0.0060 (11)
C14	0.0196 (12)	0.0228 (13)	0.0307 (14)	−0.0104 (10)	0.0010 (10)	−0.0042 (11)

### Geometric parameters (Å, º)

**Table:**

Br1—C2	1.895 (2)	N1—C9	1.482 (3)
Br2—C3	1.883 (2)	N1—C12	1.485 (3)
Br3—C4	1.892 (2)	N2—C13	1.473 (3)
Br4—C5	1.893 (2)	N2—C14	1.484 (3)
Cu1—O5	1.9744 (16)	N2—C10	1.487 (3)
Cu1—O1	1.9776 (15)	C1—C2	1.391 (3)
Cu1—N1	2.0340 (19)	C1—C6	1.402 (3)
Cu1—N2	2.0995 (19)	C1—C7	1.521 (3)
Cu1—O3^i^	2.1396 (14)	C2—C3	1.396 (3)
O1—C7	1.269 (3)	C3—C4	1.398 (3)
O2—C7	1.243 (3)	C4—C5	1.393 (3)
O3—C8	1.263 (3)	C5—C6	1.387 (3)
O3—Cu1^i^	2.1396 (14)	C6—C8	1.524 (3)
O4—C8	1.246 (3)	C9—C10	1.516 (3)
N1—C11	1.475 (3)		
			
O5—Cu1—O1	92.80 (6)	C2—C1—C7	122.73 (19)
O5—Cu1—N1	177.14 (7)	C6—C1—C7	117.98 (18)
O1—Cu1—N1	89.71 (7)	C1—C2—C3	121.13 (19)
O5—Cu1—N2	91.13 (7)	C1—C2—Br1	118.64 (16)
O1—Cu1—N2	136.68 (7)	C3—C2—Br1	120.21 (16)
N1—Cu1—N2	86.10 (8)	C2—C3—C4	119.12 (19)
O5—Cu1—O3^i^	90.46 (6)	C2—C3—Br2	120.61 (16)
O1—Cu1—O3^i^	124.17 (6)	C4—C3—Br2	120.27 (15)
N1—Cu1—O3^i^	89.26 (7)	C5—C4—C3	119.80 (19)
N2—Cu1—O3^i^	98.90 (7)	C5—C4—Br3	120.18 (16)
C7—O1—Cu1	130.25 (14)	C3—C4—Br3	120.02 (15)
C8—O3—Cu1^i^	127.37 (14)	C6—C5—C4	120.80 (19)
C11—N1—C9	109.53 (18)	C6—C5—Br4	119.17 (16)
C11—N1—C12	108.07 (19)	C4—C5—Br4	120.01 (16)
C9—N1—C12	111.12 (19)	C5—C6—C1	119.73 (19)
C11—N1—Cu1	113.14 (14)	C5—C6—C8	120.89 (19)
C9—N1—Cu1	103.09 (14)	C1—C6—C8	119.35 (18)
C12—N1—Cu1	111.86 (15)	O2—C7—O1	127.93 (19)
C13—N2—C14	108.52 (19)	O2—C7—C1	118.85 (18)
C13—N2—C10	111.32 (19)	O1—C7—C1	113.20 (18)
C14—N2—C10	110.3 (2)	O4—C8—O3	127.65 (19)
C13—N2—Cu1	110.61 (15)	O4—C8—C6	117.88 (18)
C14—N2—Cu1	109.81 (14)	O3—C8—C6	114.47 (18)
C10—N2—Cu1	106.31 (14)	N1—C9—C10	109.8 (2)
C2—C1—C6	119.26 (19)	N2—C10—C9	109.52 (19)
			
C6—C1—C2—C3	3.0 (3)	C2—C1—C6—C8	178.75 (18)
C7—C1—C2—C3	−178.78 (19)	C7—C1—C6—C8	0.5 (3)
C6—C1—C2—Br1	−175.50 (15)	Cu1—O1—C7—O2	−1.5 (3)
C7—C1—C2—Br1	2.7 (3)	Cu1—O1—C7—C1	−179.92 (13)
C1—C2—C3—C4	0.2 (3)	C2—C1—C7—O2	60.4 (3)
Br1—C2—C3—C4	178.71 (15)	C6—C1—C7—O2	−121.4 (2)
C1—C2—C3—Br2	−179.24 (15)	C2—C1—C7—O1	−121.0 (2)
Br1—C2—C3—Br2	−0.7 (2)	C6—C1—C7—O1	57.2 (2)
C2—C3—C4—C5	−3.3 (3)	Cu1^i^—O3—C8—O4	−6.9 (3)
Br2—C3—C4—C5	176.09 (16)	Cu1^i^—O3—C8—C6	172.16 (13)
C2—C3—C4—Br3	176.10 (15)	C5—C6—C8—O4	66.3 (3)
Br2—C3—C4—Br3	−4.5 (2)	C1—C6—C8—O4	−115.6 (2)
C3—C4—C5—C6	3.3 (3)	C5—C6—C8—O3	−112.8 (2)
Br3—C4—C5—C6	−176.16 (16)	C1—C6—C8—O3	65.3 (3)
C3—C4—C5—Br4	−175.18 (16)	C11—N1—C9—C10	−170.79 (19)
Br3—C4—C5—Br4	5.4 (2)	C12—N1—C9—C10	69.9 (2)
C4—C5—C6—C1	0.0 (3)	Cu1—N1—C9—C10	−50.1 (2)
Br4—C5—C6—C1	178.46 (15)	C13—N2—C10—C9	−148.7 (2)
C4—C5—C6—C8	178.09 (19)	C14—N2—C10—C9	90.8 (2)
Br4—C5—C6—C8	−3.4 (3)	Cu1—N2—C10—C9	−28.2 (2)
C2—C1—C6—C5	−3.1 (3)	N1—C9—C10—N2	54.4 (3)
C7—C1—C6—C5	178.62 (18)		

Symmetry code: (i) −*x*+1, −*y*, −*z*+1.

### Hydrogen-bond geometry (Å, º)

**Table:**

*D*—H···*A*	*D*—H	H···*A*	*D*···*A*	*D*—H···*A*
O5—H5*A*···O2	0.81 (2)	1.87 (2)	2.649 (2)	161 (2)
O5—H5*B*···O4^i^	0.82 (2)	1.83 (2)	2.630 (2)	167 (3)
C10—H10*A*···Br4^ii^	0.99	2.84	3.729 (3)	150
C11—H11*C*···O4	0.98	2.40	3.376 (3)	179
C13—H13*A*···O4^iii^	0.98	2.58	3.506 (3)	158

Symmetry codes: (i) −*x*+1, −*y*, −*z*+1; (ii) *x*−1, *y*−1, *z*; (iii) *x*−1, *y*, *z*.

## References

[bb1] Aakeröy, C. B., Beatty, A. M., Desper, J., O’Shea, M. & Valdés-Martínez, J. (2003). *Dalton Trans.* pp. 3956–3962.

[bb2] Bruker (2012). *APEX2*, *SAINT* and *SADABS*. Bruker AXS Inc. Madison, Wisconsin, USA.

[bb3] Colacio, E., Aouryaghal, H., Mota, A. J., Cano, J., Sillanpää, R. & Rodríguez-Diéguez, A. (2009). *CrystEngComm*, **11**, 2054–2064.

[bb4] Dorazco-González, A., Martínez-Vargas, S., Hernández-Ortega, S. & Valdés-Martínez, J. (2013). *CrystEngComm*, **15**, 5961–5968.

[bb5] Dorazco-González, A. & Yatsimirsky, A. K. (2010). *Inorg. Chim. Acta*, **363**, 270–274.

[bb6] Ene, C. D., Madalan, A. M., Maxim, C., Jurca, B., Avarvari, N. & Andruh, M. (2009). *J. Am. Chem. Soc.* **131**, 4586–4587. 10.1021/ja900416e 19334769

[bb7] Hong, C. S. & You, Y. S. (2004). *Polyhedron*, **23**, 3043–3050.

[bb8] Julve, M., Faus, J., Verdaguer, M. & Gleizes, A. (1984). *J. Am. Chem. Soc.* **106**, 8306–8308.

[bb9] Kozlevčar, B., Leban, I., Petrič, M., Petriček, S., Roubeau, O., Reedijk, J. & Šegedin, P. (2004). *Inorg. Chim. Acta*, **357**, 4220–4230.

[bb10] Liang, M., Liao, D.-Z., Jiang, Z.-H., Yan, S.-P. & Cheng, P. (2004). *Inorg. Chem. Commun.* **7**, 173–175.

[bb11] Mendy, J. S., Saeed, M. A., Fronczek, F. R., Powell, D. R. & Hossain, A. (2010). *Inorg. Chem.* **49**, 7223–7225. 10.1021/ic100686m PMC2919498 20690729

[bb12] Rodpun, K., Blackman, A. G., Gardiner, M. G., Tan, E. W., Meledandri, C. J. & Lucas, N. T. (2015). *CrystEngComm*, **17**, 2974–2988.

[bb13] Sheldrick, G. M. (2008). *Acta Cryst.* A**64**, 112–122. 10.1107/S0108767307043930 18156677

[bb14] Stibrany, R. T. (2009). *J. Chem. Crystallogr.* **39**, 719–722.

[bb15] Valdés-Martínez, J., Cervantes-Lee, F. & ter Haar, L. W. (1993). *J. Appl. Phys.* **74**, 1918–1921.

[bb16] Yang, S.-Y., Long, L.-S., Wu, Z.-Y., Zhan, M.-X., Huang, R.-B. & Zheng, L.-S. (2002). *Transition Met. Chem.* **27**, 546–549.

