# Supplementary figures and images for: Crystal structure of bis­(μ2-tetra­bromo­phthalato-κ2 O 1:O 2)bis[aqua(N,N,N′,N′-tetra­methyl­ethane-1,2-di­amine-κ2 N,N′)copper(II)]
Source: Acta Crystallogr E Crystallogr Commun. 2015 Aug 22;71(Pt 9):m171–2. doi: 10.1107/S2056989015015194 (PMC4555376; doi:10.1107/S2056989015015194)

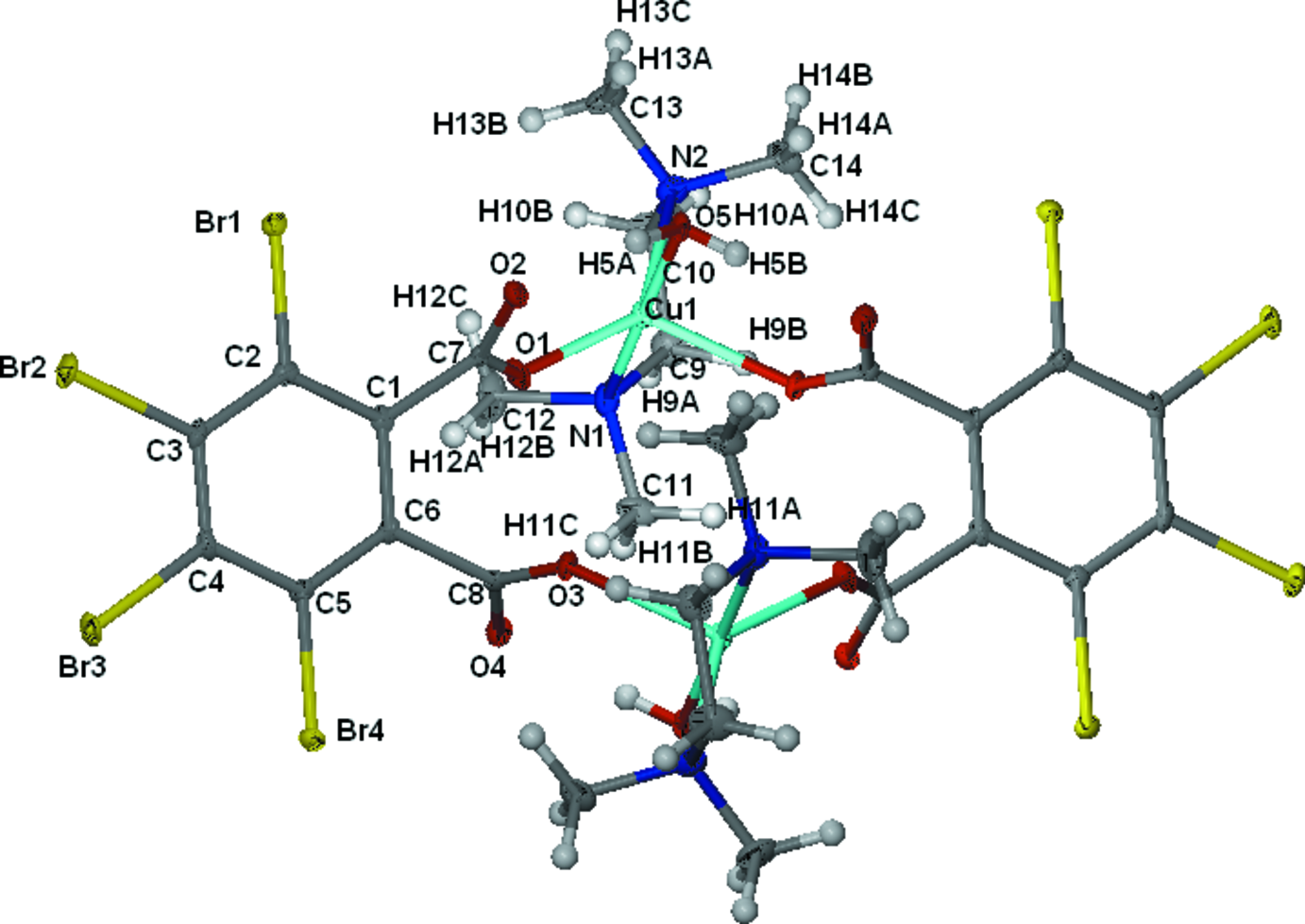

Supplement: Supplementary file 3 [file e-71-0m171-fig1.tif]

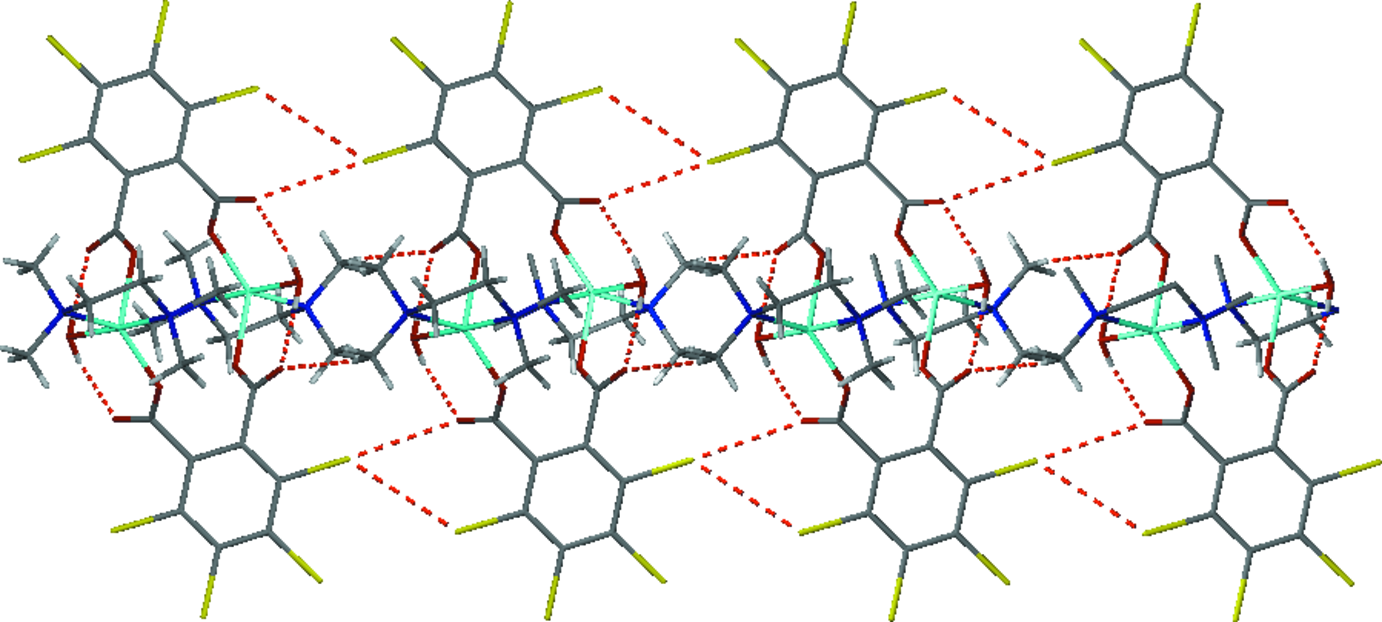

Supplement: Supplementary file 4 [file e-71-0m171-fig2.tif]
